# Proprioception and its relationship with range of motion in hypermobile and normal mobile children

**DOI:** 10.1007/s00221-024-06937-1

**Published:** 2024-10-08

**Authors:** Oluwakemi A. Ituen, Bouwien Smits-Engelsman, Gillian Ferguson, Jacques Duysens

**Affiliations:** 1 Department of Health & Rehabilitation, University of Cape Town, Cape Town, South Africa; 2 University of Uyo Teaching Hospital, Uyo, Akwa Ibom State Nigeria; 3 Physical Activity, Sport and Recreation, Faculty Health Sciences, North-West University, Potchefstroom, South Africa; 4 Motor Control Laboratory, Movement Control and Neuroplasticity Research Group, KU Leuven, Leuven, Belgium

**Keywords:** Hypermobility, Children, Proprioception, Joint loading

## Abstract

To investigate differences in proprioception using four proprioceptive tests in children with and without hypermobility. Additionally, it was tested if the results on one proprioceptive test predict the results on the other tests. Of the children (8-11years), 100 were classified as normal mobile (Beighton score 0–4) and 50 as hypermobile (Beighton score 5–9). To test proprioception, in the upper extremity the unilateral and bilateral joint position reproduction tasks were used and for the lower extremity the loaded and unloaded wedges task. No differences were found in any of the proprioception tests between the two groups. Estimating the height of the wedges was easier in the loaded position (mean penalty in standing and sitting position, 4.78 and 6.19, respectively). Recalling the elbow position in the same arm resulted in smaller errors compared to tasks reproducing the position with the contralateral arm. Of the four angles used (110°, 90°, 70°, 50°), the position recall in the 90° angle had the smallest position error (1.8°). Correlations between the proprioception tests were weak (Loaded and Unloaded (r 0. 28); Uni and Bilateral (r 0.39), Upper and Lower extremity not significant). No indication of poorer proprioception was found in children with hypermobile joints compared to their normal mobile peers. Loading gives extra information that leads to fewer errors in the wedges task performed while standing, but this effect is independent of joint mobility. Proprioception test outcomes are dependent on the test used; upper extremity results do not predict lower extremity outcomes or vice versa.

## Introduction

If proprioception depends on joint mobility one would predict worse proprioception in hypermobile children. Joint Hypermobility (JH) is the ability of a joint to move beyond the normal range of motion as a result of laxity of ligaments (Castori and Hakim 2017). When it occurs in multiple joints it is referred to as Generalized Joint Hypermobility (GJH) (Przymuszała et al. 2018). Its prevalence in the general population usually is influenced by age, gender and race (Kwon et al. 2013). The Beighton scoring system has been commonly used to identify children with GJH (Malek et al. 2021). The prevalence of GJH has varied between 8.8% and 64.6% in literature partly because of the lack of consensus on the Beighton score cut off (Ituen et al. 2023). Previous studies have used Beighton score cut-offs ranging from ≥ 3 to ≥ 6 (Reuter and Fichthorn 2019; Kurniawati et al. 2021).

GJH is a physical trait that is viewed as the end of the normal spectrum of joint mobility and it is predominately asymptomatic; it is perceived to be beneficial in motor activities that require flexibility (Tofts et al. 2009; Van Meulenbroek et al. 2020). In contrast, GJH may also represent a polygenic group of a mild end of the spectrum of the Heritable Disorders of Connective Tissues (HDCT) because of its association with musculoskeletal symptoms such as pain, sprains, impaired proprioception, reduced muscle strength, and poor motor coordination (Engelbert et al. 2017; Maarj et al. 2023). This is referred to as Hypermobility Spectrum Disorder (HSD). However, not all children with GJH become symptomatic and the evidence on the role joint laxity plays in the pathology of musculoskeletal symptoms in literature has not been conclusive (Simmonds and Keer 2007).

Proprioception is the awareness of the body in space and it is essential in motor control and coordination (Virginia 2017). Children with GJH have been described as clumsy with associated impaired proprioception (Akkaya et al. 2023). However, gap persists in the literature about possible proprioception deficits in children with hypermobility without musculoskeletal complaints because previous authors have tested proprioception using different instruments and test positions (Smith et al. 2013). Hence there is a need to retest the potential proprioceptive deficit in these children.

In an earlier study, we used wedges to test proprioception in the lower extremity in children with GJH in a weight-bearing position and no difference was detected compared to controls (Ituen et al. 2020). In a more recent study, we modified the heights of the wedges making the test more difficult, yet it did not reveal a difference between children with and without GJH (Anieto et al. 2023). It is yet to be tested if the outcomes will be different when the wedge test is performed in unloaded positions. Given the additional input from load receptors in the loaded condition one expects better performance in the loaded condition as compared to the unloaded one. Furthermore, it can be argued that the absence of difference between mobility groups in our previous studies could be due to the choice of the lower extremity while most studies used upper extremity tasks. The most frequently used upper extremity tests are the unilateral and bilateral elbow position matching tasks Therefore, we administered both upper and lower extremity tasks in children with and without hypermobility.

Peculiar to joint hypermobility is the abnormal joint biomechanics arising from the laxity of the joint connective tissues (Pacey et al. 2010). It is assumed that the demand to maintain joint stability may put some strain on the connective tissues causing repetitive micro and macro trauma over time (Tinkle 2020). The long-term effect may be a gradual destruction of mechanoreceptors at the joints and deficits in their function (Anieto et al. 2023). Mechanoreceptors are located in the joint capsular tissues, ligaments, tendons, muscle, and skin tissues and perform the function of passing sensory stimuli to the central nervous system for processing, resulting in appropriate motor responses (Ageberg et al. 2007). Appropriate functioning of these mechanoreceptors is important for the role of proprioception in motor performance and physical activities (Han et al. 2016).

The aims of the present study were firstly to compare the outcomes on proprioception in a comprehensive set of tests (four different target angles in the upper limbs and wedges in loaded and unloaded positions in lower limbs between children with and without GJH). Secondly, we investigated whether the loaded wedges task was superior to the unloaded task and if the results in one proprioceptive test predicted the results in the other tests.

## Materials and methods

### Procedure

The study used a cross-sectional descriptive design and was conducted following the Declaration of Helsinki. The study’s ethical approval was obtained both from the human research ethics committee of the University of Cape Town (UCT HREC: 096/2015, HREC REF: 306/2021) and the University of Uyo Teaching Hospital REF: UUTH/AD/S/96/VOL/ XXI/524. The secretary of the Local Government education Uyo, along with the head teachers and class teachers at the selected schools, all granted permission to carry out the test on the children. Schools were selected through the convenience sampling method. Our study exclusion criteria were: children with cognitive and gross motor impairment, as reported by their parents, because these limitations would affect their ability to understand the testing instructions or their performance of the activities. However, none of the children had to be excluded using the above criteria. The study sample size was calculated through a power analysis that showed that a total sample size of 134 was needed for a medium effect size (d = 0.6), at a power of 90%, while alpha was set at 0.05 with an allocation ratio of 2. The G-power analysis software version 3.1.9.2 was used for the sample size calculation (Faul et al. 2007). Written informed consent was obtained from the parents or legal guardians of the children, and assent was given by the children before their enrolment. The children were tested by seven trained researchers in their school. The children were given breaks between tests or at their request.

### Demographic measures

Data were collected on participants’ age (years), sex, height (centimeters), and weight (kilograms). Height and weight were measured using measuring tape and a weighing scale (on bare feet; measured to the closest one cm and 100 g, respectively). The body mass index (BMI) calculation was performed using a metric formula, weight (in kilograms) divided by height (in meters squared).

### Beighton score

The nine-point Beighton score, with goniometry, was used to assess joint mobility (Ituen et al. 2023). The test consists of bilateral assessment of the 5th metacarpophalangeal (MCP), elbow, knee joints, thumb movement and one active forward trunk flexion task. A score of 0–9 was used to divide joint mobility into two categories, normal mobility (0–4) and hypermobility (5–9) (Nikolajsen et al. 2013). The Beighton test has been validated among children (Smits-Engelsman et al. 2011).

### Experimental protocol

For the upper extremity proprioception test, the joint position reproduction (JPR) using a goniometer was used as the method of measuring position sense by repositioning. We tested elbow JPR unilaterally and bilaterally, randomizing the order of tasks and angles in both experiments. The tests were carried out with the child seated, arms by the side and blindfolded. Starting with either the preferred (four trials) or the non-preferred side (four trials), the order of test angles was random. A first tester passively moved the arm in the coronal plane (arm to the side with the shoulder at about 90°), then moved the elbow joint to the target angle (50°,70°,90°,110°) and the child was asked to concentrate on the angle while the tester maintaining this position for 10s. Then the tester dropped the hand by the side of the child. The child was then asked to reposition the elbow without delay to the detected angle, and the measurement was taken with a goniometer by the second tester. The convention was used that 0° represented a fully extended arm with the forearm horizontal, and the 90° position was a flexed arm with the forearm in the vertical position. The difference between the target angle and the child’s test result was determined as the error score. Half of the children started the trials with the dominant hand and the other half with the non-dominant hand. Three measurements were made for all target angles, and the arithmetic mean of the differences was calculated.

### Unilateral (ipsilateral) joint position reproduction task

The tester presented four target angles to the right and left arm, thus subjects performed 8 ipsilateral matching trials (with 3 repetitions). The right or the left elbow was passively moved from the starting position to one of the four target angles earlier described. After the tester returned the arm to the baseline position, subjects then repositioned the same arm to the target angle and the tester measured with the goniometer.

### Bilateral (contralateral) joint position reproduction task

The tester passively moved the elbow to one of the same four angles as mentioned in the experimental protocol for the unilateral task. The subject was then asked to position the contralateral arm in the target angle. The other tester measured the angle of the contralateral arm with the goniometer. The tester presented four target angles thus subjects performed 8 contralateral matching trials (with 3 repetitions).

### Wedges test

Using different wedges under the feet the ankle angle can be varied, and the perceived height can be measured. This can be done in weight-bearing (loaded) position (Ituen et al. 2020) but here it is also assessed in non-weight-bearing conditions.

### Loaded task

We tested proprioception (detection of heel-height difference) using the wedges of various heights that produce different angles equal to contact surface of 1.5°, 3°, 0.4.5°, 6°, 9° and 12°. The 1.5°, 4.5° wedges were added (similar to Anieto et al. 2023) to have more combinations with only 1.5° difference (1.5° versus 3°, 3° versus 4.5°, 4.5° versus 6°). Participants stood behind a table and were not blindfolded during the testing but were instructed not to look at their feet under the table while the test was conducted. While standing on the wedges, (without support from the table) they raised the arm of the side with the higher ankle, for example, the right arm for the right ankle. Both arms were raised when no difference in ankle height was detected. The subject had 5s to respond. A penalty score was awarded for every incorrect response, and the size of the penalty was determined by differences in the height of the wedges. The higher the wedge height difference, the higher the penalty score. The individual penalty scores of the 21 test trials were summed up to get a total penalty; a high penalty score indicates poor proprioception.

### Unloaded task

The unloaded version of the test was carried out with the participants seated on a chair with the back rested against a wall to minimize the weight on the leg during the test and their knees were positioned at 90°. With eyes closed, the wedges were placed under the feet of the participants, and they were asked to raise the hand on the side of the ankle with the higher ankle. Both arms were raised when no difference in ankle height was detected. The subject had 5s to respond.

### Statistical analysis

All variables were examined to determine whether distributions were normal or skewed. No outliers were present in the data, except for 2 data points in the loaded wedges task, which were removed. T-tests were used to test for differences in demographic variables between the two groups and Chi^2^ to test the gender distribution over groups.

A GLM Repeated Measures was used to examine the effect of JPR tasks (Uni and Bilateral) hand (Preferred and Non-preferred), angle (110°, 90°, 70°, 50°) as within-subject factors and group (Hypermobile, Normal mobile) as between-subject factor and to test for possible interactions. A second GLM Repeated Measures was used to examine the effect of loading in the wedges task (Loaded and Unloaded) as within-subject factors and group (Hypermobile, Normal mobile) as between-subject factor. Post hoc tests with Bonferroni correction were used if interactions were found. Pearson’s correlations were conducted to assess the relationships among the proprioception test performances. The significance level was set at *p* < 0.05. All statistical analyses were conducted using the statistical package for the social sciences software (SPSS, version 29.0, SPSS Inc., Chicago, IL, USA).

## Results

### Demographic data

A total of 150 children were included in the study, eighty-five (57.3%) of the children were girls and 64 (42.3%) were boys. Demographic data such as age, height, body weight, and BMI of children with and without GJH were not different (*p* > 0.05) (Table 1). Participants were classified as GJH with a Beighton score ≥ 5/9.

**Table 1:** Demographic characteristics of the participants

Demography	Children with GJH *n* = 50 mean ± SD	Children without GJH *n* = 100 mean ± SD	Total group *n* = 150 mean ± SD	*p*-value
Age (years)	9.26 ± 0.89	9.46 ± 0.83	9.39 ± 0.86	0.674
Weight (kg)	28.32 ± 6.62	27.14 ± 5.78	27.53 ± 6.07	0.214
Height (cm)	134.58 ± 8.38	134.10 ± 8.46	134.26 ± 8.41	0.473
BMI (kg/m^2^)	15.50 ± 2.60	15.02 ± 2.45	15.18 ± 2.50	0.136

GJH = Generalized Joint Hypermobility, kg = kilogram, BMI = Body Mass Index, cm = centimeter, m = meter, n = number of participants

### Joint position reproduction tasks

An overview of means and confidence intervals are given in Table 2.

**Table 2:** Means (SE) of the errors (in degrees) for the two mobility groups of all the conditions of the joint position reproduction tasks

	Mean	Standard error	95% Confidence Interval	
			Lower Bound	Upper Bound
Mobility group				
GJH Without GJH				
Body side				
Preferred Non-preferred	5.56 5.29	0.27 0.23	5.04 4.83	6.09 5.75
Target angle				
110° 90° 70° 50°	4.30 1.84 7.34 8.22	0.24 0.16 0.35 0.53	3.83 1.54 6.66 7.17	4.76 2.15 8.03 9.27
Task				
Unilateral Bilateral	4.34 6.51	0.21 0.29	3.92 5.93	4.76 7.09

GJH = Generalized Joint Hypermobility

### Main effect of group: hypermobile versus normal mobile

No group differences were found in the position errors on any of the tasks between groups (F1,147 = 0.00, p 0.992). Notably, no interactions with Task, Angle or Arm emerged with group. This indicates that children with and without hypermobility responded similarly to the task conditions.

### Main effect of side: preferred /non-preferred

**Fig. 1:**
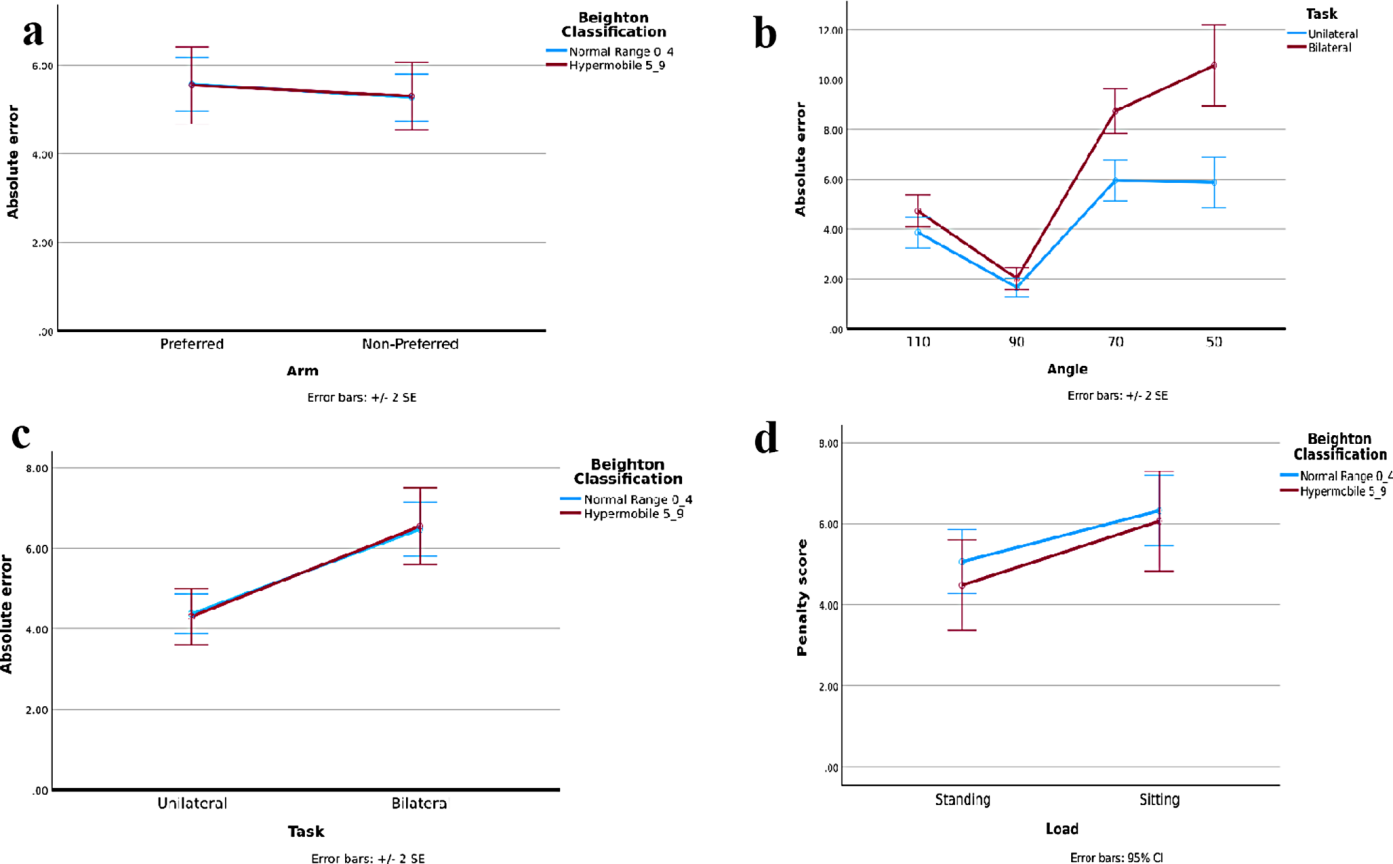
Means (SE) of the errors (in degrees) for the two mobility groups of all the conditions of the Joint Position Reproduction tasks. **a** Main effect of side (preferred /non-preferred hand) in the Joint Position Reproduction tasks. **b** Interaction effect of angle and task on the Joint Position Reproduction tasks. **c** Effect of unilateral and bilateral arm on the Joint Position Reproduction tasks. **d** Main effect of loading on the wedges

No main effect of arm was found (F(1,147) 1.090, *p* = 0.298). Although the mean error for the non-preferred arm seems lower (better performance) this was far from being significant (5.56° and 5.29 ° (see Fig. 1a), respectively for the Preferred and Non-Preferred arm (*p* = 0.289).

### Main effect of target angle: 110°, 90°, 70°, 50°

A large main effect of the angle occurred (F(3,145) 106.75, p ˂ 0.001). In the 90° angle, the position recall had the smallest position error (1.8*°)*. The more flexed angles in the elbow were clearly harder to match 7.3° and 8.2° errors for the 70° and 50° angles, respectively as shown in Fig. 1b. Post hoc showed significant differences between all angles except between 50 and 70°.

### Main effect of task: unilateral versus bilateral joint position reproduction tasks (UNI/BI)

The error was different in the recall of the position in the same arm (UNI) compared to putting the contralateral arm at the same angle as the reference arm (BI) (F(1,147) 57.36, p ˂0.001). Children were better at reproducing the position in the unilateral (error 4.3°) than the bilateral matching task (error 6.5°), as shown by the smaller absolute error (Fig. 1c). Here, the only interaction in the analysis emerged: task (Uni/BI) by angle (F(3,145) 10.76, *p* = 0.001). Post hoc showed no effect of task (Uni/BI) in the 90-degree angle. It also showed that the negative effect in the bilateral task is larger in the more difficult angles (70° and 50°) (Fig. 1b). In the bilateral task, all angles were significantly different, while in the Unilateral 50 and 70 degrees the errors were not different from each other.

### Wedges

No differences between hypermobile and normal mobile children on penalty score were found (F (1,148) 0.15, *p* = 0.70). The height discrimination results of children with and without GJH were similar (*p >* 0.05). Importantly, an effect of loading was shown (F (1,148) 11.89; *p* < 0.001; η^2^_p_ 0.074; Fig. 1d), indicating that discriminating between the height of the wedges was easier in standing than sitting (mean penalty in standing and sitting position, 4.78 and 6.19, respectively).

## Associations between the different proprioception tests

The correlation between the penalty score Loaded and Unloaded was (*r* = 0. 28, *p* < 0.001) between Uni and Bilateral position errors (r 0.39, *p* < 0.001). Because it was expected that the mobility of the elbow (range of motion) might have an impact on the two position-matching tasks, the correlation between these measurements was also determined. Similarly, the correlation between the knee and ankle mobility and the Wedges outcomes were also examined. Elbow range of motion was not significantly related to the position sense tasks. Knee and ankle range of motion showed no significant relation with loaded or non-loaded wedges results. The correlations between upper and lower extremity proprioception items were not significant.

## Discussion

The first aim of the present study was to examine whether the earlier finding of an absence of a proprioceptive deficit in GJH remained valid when broader testing was applied, that is wedges test in loaded and unloaded positions in lower limbs (Ituen et al. 2020; Anieto et al. 2023), along with elbow joint position tests. The results show that none of the added tests revealed a significant difference between GJH and controls, thereby reinforcing the idea that proprioception (as tested with a broad range of tests) is not affected in asymptomatic GJH. These findings are in line with those of Pacey et al. 2014. They studied children with more severe disease (symptomatic joint hypermobility), yet found no evidence for altered proprioception, similar to our findings on asymptomatic GJH.

These are important results since previous studies had suggested that a proprioceptive deficit existed and justified a program of proprioceptive training (Fatoye et al. 2009). However, the latter study was conducted by testing knee position sense on hypermobile children with painful knees. Another study based on 20 hypermobile children, concluded that there was a proprioceptive deficit but they only used ankle reposition at a single flexion (15°) and extension angle (25°) (Akkaya et al. 2023). Hence overall the evidence for a proprioceptive deficit is weak.

From an absence of proprioceptive deficit one would predict that physical activity is relatively normal in this hypermobile group. Indeed, no difference in daily level and duration of physical activity was observed in a group of 8-year-old school children with generalized joint hypermobility (Juul-Kristensen et al. 2009). A possible factor is that there is an element of compensation. For example, there is evidence that GJH children use a different muscle co-activation strategy to stabilize joints (Jensen et al. 2013). In addition, physical condition may be a crude parameter and finer testing may reveal deficits. For example, Anieto et al. (2023) found an association between joint mobility and motor performance, as measured with the PERF-FIT).

### Is the wedge test a valid instrument?

A possible first objection to the previous use of the wedge test was that it was performed under loaded conditions, allowing a major contribution of load receptor input, in contrast to most joint position tests. It was indeed found that the loaded test yielded better results (smaller errors) than the unloaded version. However, the unloaded version did not reveal a performance difference between the two groups investigated, thereby eliminating the presence or absence of load as a factor in the absence of a proprioceptive deficit. Several elements could contribute to the superiority of the loaded version, including the addition of load receptor input (Gooey et al. 2000) and a role of central command (Proske and Gandevia 2009). Inversely, the inferiority of the unloaded version could be related to the leg muscles being relaxed, leading to a greater likelihood of a thixotropic effect (Proske et al. 1993). Such weight-bearing superiority was also observed in knee joint matching tests as described by Stillman and McMeeken (2001), although in their case the situation was more complex as the subjects were able to use cues during movement of the knee between positions (Stillman and McMeeken 2001). In addition, these authors found no clinically significant correlation between the weight-bearing and non-weight-bearing results, confirming our present results.

Another objection, which could be used against our previous results, was that they all involved the lower limb while joint position tests are more commonly performed with the arms. The presently added arm tests again failed to show differences between the two groups. Furthermore, the correlations between arm and leg tests were not significant. This is not surprising as a recent review on the topic concluded that results with respect to one body part may not be generalized to others (Horvath et al. 2023). The current tests were performed on each arm separately and it was found that the mean error for the non-preferred arm was slightly lower than for the preferred arm. Although not significant, this result is in line with current literature (Goble et al. 2006) We also compared unilateral (one-arm repositioning) and bilateral (two-arm matching) elbow joint position tests and found bilateral tests to yield worse results than unilateral ones. This could have been expected since many more computations are required in bilateral testing. For future work it would be of interest to have a bilateral condition without repositioning but with different angles on both sides to make subjects decide which side was most extended (in analogy with the wedges test).

For the unilateral matching, it was of special interest that the error depended on arm position (angle). For both the unilateral and the bilateral testing the optimum was reached for elbow angles around 90 degrees (hence forearm in a vertical position). This is similar to the findings of Roach et al. (2023), who found that the smallest repositioning errors were seen at an elbow angle near 90 degrees (Roach et al. 2023). What is so special about holding the lower arm in an upright vertical position? Stretch of muscle spindles, skin or joint receptors is higher at other angles. Torque sensation is likely to be at a minimum at 90 degrees and should not be overlooked as an accessory source of information in limb positioning (Worringham and Stelmach 1985). In addition, there may be a perceptual component. In the visual system, it is known that vertical and horizontal line orientations are best for orientation discrimination (“oblique effect”) (Orban et al. 1984). This bias persist even when the head is tilted, thereby suggesting that it is mostly an allocentric effect (Mikellidou et al. 2015). More generally, a crucial factor is the “subjective vertical”, the perception of verticality (Dakin and Rosenberg 2018), When subjects are asked to judge elbow position with respect to the vertical they are better than when focusing on joint angle (Soechting 1982). The latter author concluded that position sense at the elbow joint includes a sense of the orientation of the forearm with respect to a spatial frame of reference, while the elbow joint angle is only imperfectly sensed. The question arises which afferents contribute to the normal responses in the hypermobile population. Could joint receptors be involved? If laxity of ligaments is a problem, then it would be expected that torque at the joint would be reduced and this would lead to a fall in joint receptor input, especially at the limits of the movement range at the joint. Therefore, it is more likely that muscle spindles are the main sensors involved. This is in line with the general notion that spindle afferents (especially the Ia afferents) are the principal contributors to position sense (Goodwin et al. 1972).

Returning to our main result and the evidence supporting the notion of an absence of a proprioceptive deficit in asymptomatic GHS, the question remains whether some subtle proprioceptive deficiencies exist, not captured by our tests. According to Heroux et al. (2022), proprioceptive tests can be classified as either a low-level or a high-level task. Repositioning is seen as low-level by some (Heroux et al. 2022) because a single frame of reference is used but others consider this to be a high-level task since higher functions (such as memory) are involved (Roach et al. 2023). Nevertheless, it is only one form of high-level testing, while others are available, for example tests in which subjects are required to indicate where their unseen hand is.

Motion sense would also be of interest to measure since it potentially involves a different set of afferent input (Krewer et al. 2016). Joint position sense and movement detection were shown to correlate poorly and therefore both are worth examining to obtain a more complete picture of proprioception (Grob et al. 2002). However, so far no evidence was found for deficiencies in more dynamic conditions as we found no differences between a GJH group and a control group on the Y-balance performance, a test that requires a reach movement with the free foot while standing on one leg (Ituen et al. 2024).

In hypermobile people with Ehlers-Danlos syndrome (EDS), joint angle matching does not reveal any difference with controls (similar to the present study on generalized hypermobility). In contrast, a hand position test showed the hypermobile EDS subjects to be less accurate than controls (Clayton et al. 2021). This emphasizes the complexity of the notion of proprioception and points to potential follow-up studies, using hand position testing, on GJH children.

## Conclusion

The current data show that there is no evidence for a proprioceptive deficit in children with generalized hypermobility as tested with a broad set of proprioceptive tests.

## Data Availability

The datasets used and/or analyzed during the current study are available from the corresponding author on reasonable request.

## Abbreviations

NM: Normal Mobility
HM: Hypermobility
BMI: Body mass index
GJH: Generalized Joint Hypermobility
HSD: Hypermobility Spectrum Disorder
RT: Right
LT: Left
JPR: Joint Position Reproduction
HDCT: Heritable Disorders of Connective Tissues
JH: Joint Hypermobility
MCP: Metacarpophalangeal
